# GSK3 coordinately regulates mitochondrial activity and nucleotide metabolism in quiescent oocytes

**DOI:** 10.1242/bio.061815

**Published:** 2025-03-24

**Authors:** Leah Eller, Lei Wang, Mehmet Oguz Gok, Helin Hocaoglu, Shenlu Qin, Parul Gupta, Matthew H. Sieber

**Affiliations:** ^1^ UT Southwestern: The University of Texas Southwestern Medical Center, USA; ^2^ UT Southwestern Medical Center, Department of Physiology, 5323 Harry Hines Blvd., Dallas, Texas 75390, USA

**Keywords:** *Drosophila*, Oocyte, Embryo, Metabolism, Mitochondria

## Abstract

As cells transition between periods of growth and quiescence, their metabolic demands change. During this transition, cells must coordinate changes in mitochondrial function with the induction of biosynthetic processes. Mitochondrial metabolism and nucleotide biosynthesis are key rate-limiting factors in regulating early growth. However, it remains unclear what coordinates these mechanisms in developmental systems. Here, we show that during quiescence, as mitochondrial activity drops, nucleotide breakdown increases. However, at fertilization, mitochondrial oxidative metabolism and nucleotide biosynthesis are coordinately activated to support early embryogenesis. We have found that the serine/threonine kinase GSK3 is a key factor in coordinating mitochondrial metabolism with nucleotide biosynthesis during transitions between quiescence and growth. Silencing GSK3 in quiescent oocytes causes increased levels of mitochondrial activity and a shift in the levels of several redox metabolites. Interestingly, silencing GSK3 in quiescent oocytes also leads to a precocious induction of nucleotide biosynthesis in quiescent oocytes. Taken together, these data indicate that GSK3 functions to suppress mitochondrial oxidative metabolism and prevent the premature onset of nucleotide biosynthesis in quiescent eggs. These data reveal a key mechanism that coordinates mitochondrial function and nucleotide synthesis with fertilization.

## Teaser

**Summary:** The serine-threonine kinase GSK3 coordinates mitochondrial activity and nucleotide metabolism in quiescent oocytes.

## INTRODUCTION

During development, a cell's metabolic and physiological demands change dramatically (Sieber and Spradling, 2017; Tippetts et al., 2023). Changes in cellular behavior, activity, signaling, and fate can alter the metabolic pathways required to maintain health and normal function (Alaynick et al., 2007; Zheng et al., 2016; Tennessen et al., 2014). Energy-producing processes like mitochondrial respiration must be coordinated with biosynthetic processes in precise stages of development (Sieber and Spradling, 2017; Tippetts et al., 2023). As a result, organisms rely on fundamental signaling mechanisms to coordinate changes in metabolism with developmental timing (Sieber and Spradling, 2015; Tennessen et al., 2011; Yue et al., 2022). However, it remains unknown how energy production and biosynthesis are coordinated in most developmental transitions.

During late *Drosophila* oogenesis, egg chambers transition from active growth to a dormant state of quiescence. During quiescence, transcription and translation decrease dramatically (Lovett and Goldstein, 1977; Mermod et al., 1977). Shutting down these processes in growing cells can reduce energetic and biosynthetic demands by over 70% (Shore and Albert, 2022). Quiescent cells also display a 90% reduction in mitochondrial respiration called mitochondrial respiratory quiescence (MRQ), which protects cells from oxidative damage and prevents the depletion of stored nutrients (Yue et al., 2022; Hocaoglu et al., 2021; Sieber et al., 2016). These changes in physiology and metabolism are highly conserved aspects of quiescent cells from yeast to humans (Sieber et al., 2016). The oocytes stay dormant until fertilization, when translation (Kronja et al., 2014) and mitochondrial activity are reactivated during early embryogenesis (Leatherman and Jongens, 2003; Van Blerkom, 2011). This initiation of mitochondrial function in early embryos drives a significant shift in cellular redox balance (Petrova et al., 2018; Du et al., 2023). As mitochondrial activity is restored, the biosynthetic demands for building blocks such as nucleotides increase significantly. Nucleotide synthesis is required in flies for the early stages of embryonic development (Djabrayan et al., 2019). Studies have shown that nucleotides are limiting for growth in many organisms (Samant et al., 2008; Diehl et al., 2022; Mullen and Singh, 2023; Chi et al., 2016), so coordinating changes between mitochondrial metabolism and nucleotide synthesis is essential to drive rapid growth during embryogenesis. However, little is known about how mitochondrial function is coupled to nucleotide metabolism in quiescent cells.

Previously, we showed that in response to a drop in insulin/Akt signaling, GSK3, a highly conserved direct target of Akt, triggers MRQ and promotes metabolic dormancy in mature oocytes (Sieber et al., 2016). GSK3 does this by phosphorylating VDAC and other mitochondrial outer membrane proteins to promote proteasome recruitment to the mitochondrial surface (Yue et al., 2022). We have found that proteasome recruitment supports the remodeling of the mitochondrial proteome and MRQ by aiding in the turnover of outer membrane proteins (Yue et al., 2022). Given this role in oocyte quiescence, we hypothesize that GSK3 may coordinate mitochondrial function and nucleotide metabolism during transitions between quiescence and growth.

Here, we show that MRQ is coupled to the breakdown of nucleotides during quiescence in *Drosophila* oocytes. Moreover, after fertilization, when mitochondria are reactivated from their dormant state, oocytes display increased nucleotide levels, suggesting elevated nucleotide biosynthesis and reduced nucleotide breakdown. Interestingly, we have found that inactivating GSK3 in quiescent oocytes leads to changes in redox metabolism and a premature elevation in nucleotide levels, suggesting a precocious onset of nucleotide biosynthesis. These data indicate that GSK3 triggers MRQ and suppresses oxidative metabolism while preventing nucleotide biosynthesis that normally occurs after fertilization. Our data suggests that GSK3 is key in coordinating mitochondrial function and nucleotide metabolism between growth and quiescence.

## RESULTS

**Fig. 1.**
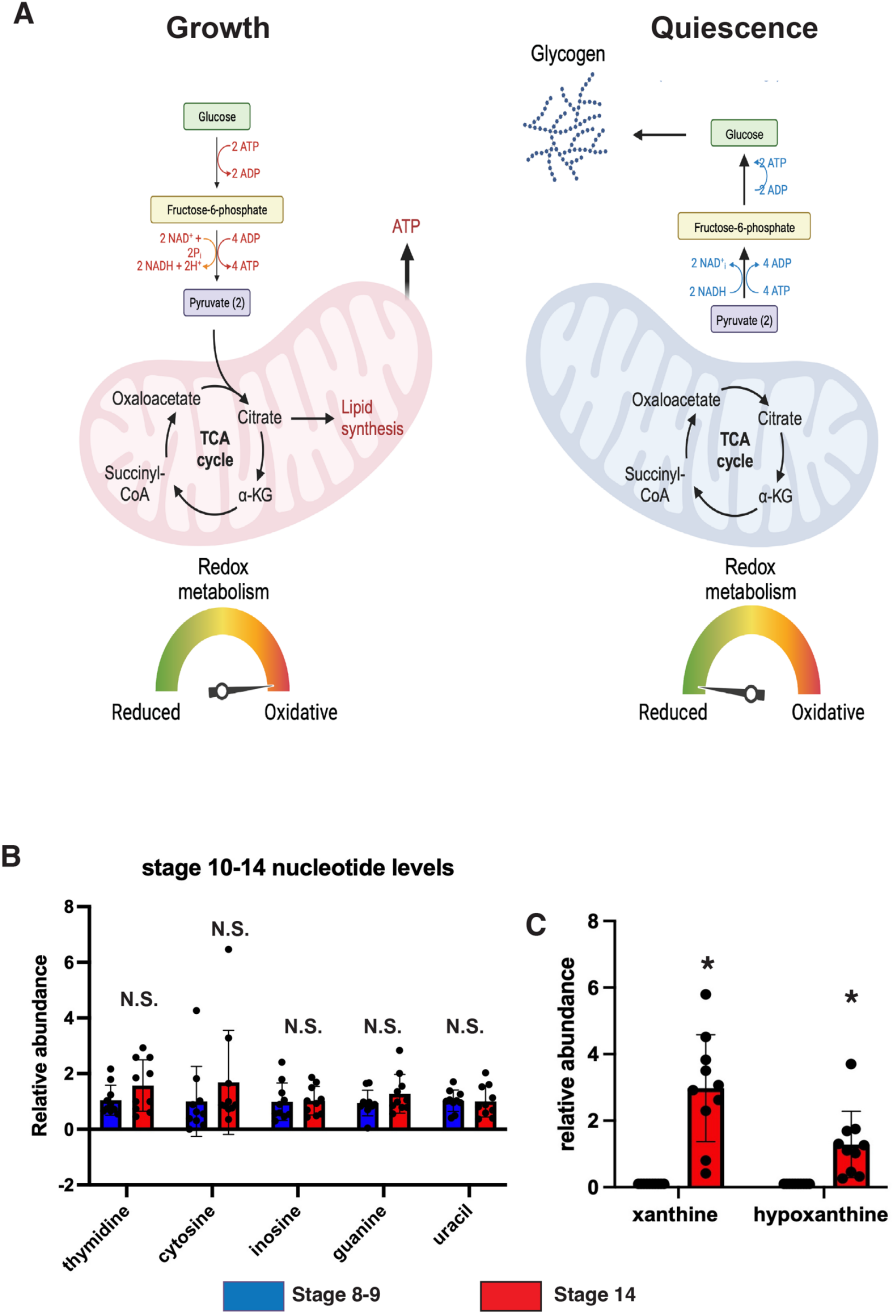
**Purine salvage pathway intermediates increase during MRQ.** (A) A model summarizing the changes in mitochondrial metabolism that occur during the transition between growing egg chambers (stage 8-9) and quiescent oocytes. (Oregon R). (B) LC/MS measurements of detectable nucleotides in growing egg chambers (stage 8-9) and stage 14 oocytes. (*n*=10 samples). (C) LC/MS measurements of nucleotide breakdown intermediates in growing egg chambers (stage 8-9) and quiescent stage 14 oocytes (*n*=10 samples). *P<0.05. *P*-values were calculated by Student’s *t*-test. Error bars represent standard deviation.

During late oogenesis, mitochondrial function and morphology are remodeled as the egg chamber transitions from active growth to quiescence (Sieber et al., 2016). In stage 10 of oogenesis, mitochondrial membrane potential is lost, glycolysis is reduced, and electron transport chain activity is suppressed in a process called MRQ. This loss of mitochondrial activity alters oocyte redox balance and promotes nutrient storage by reducing nutrient usage (Fig. 1A). Once mitochondrial activity is suppressed, oocytes remain in this dormant state until fertilization and the initiation of embryogenesis. To resume embryonic growth, mitochondrial activation must be coupled to the activity of biosynthetic pathways, such as nucleotide biosynthesis, to drive development. We examined nucleotide levels in growing egg chambers (stages 8-9) and mature quiescent oocytes by dissecting ovaries and isolating staged oocytes for LC/MS measurements of nucleotide levels. We found that levels of detectable nucleotides were unaffected during this transition (Fig. 1B). However, by-products of purine degradation were significantly elevated in stage 14 oocytes, suggesting nucleotide breakdown is elevated during MRQ (Fig. 1C). These data support the idea that nucleotide metabolism is coordinated with dynamic changes in mitochondrial activity.

**Fig. 2.**
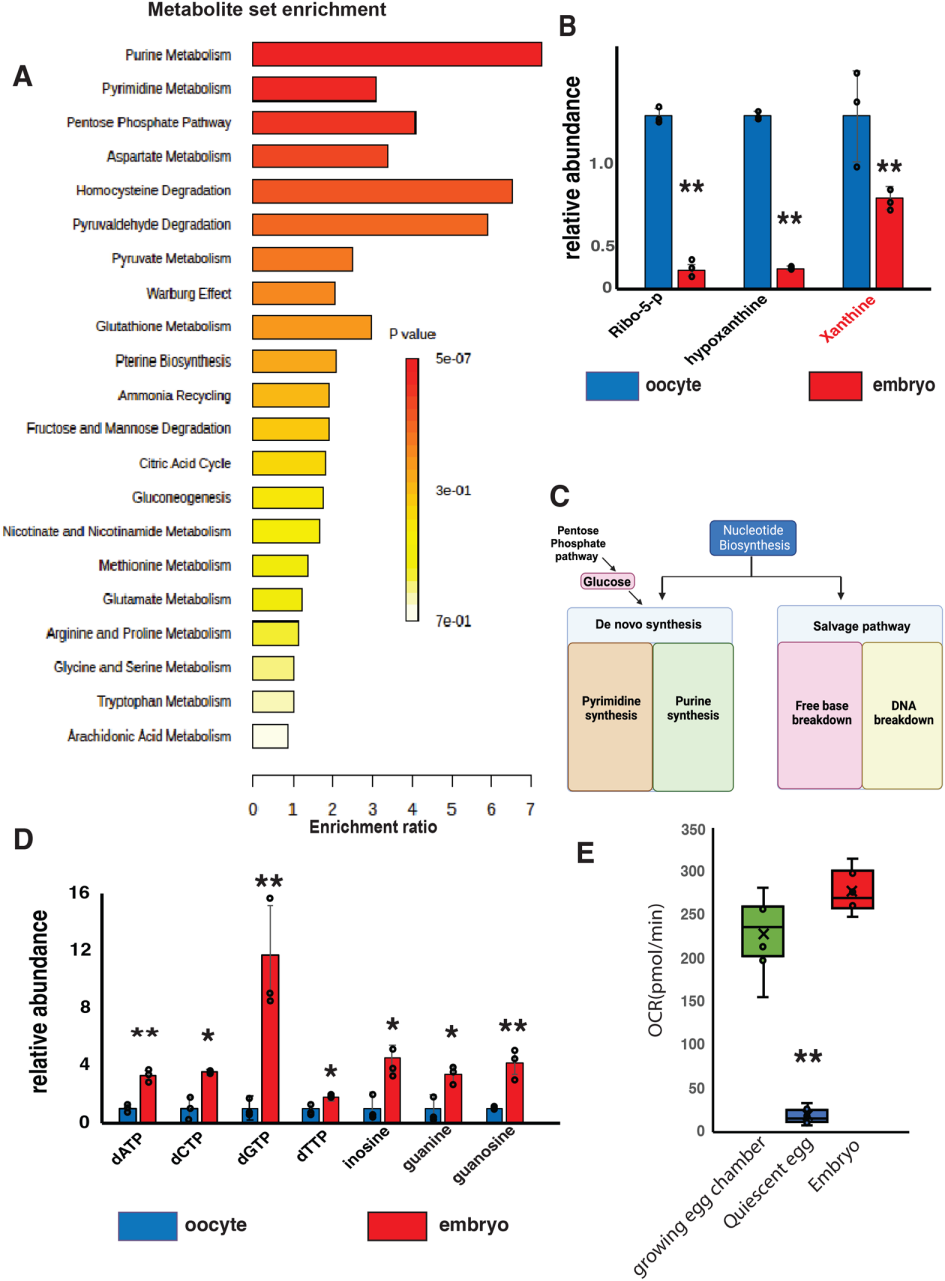
**During embryogenesis nucleotide biosynthesis increases as mitochondria are reactivated.** (A) Metabolic pathways enrichment analysis for quiescent stage 14 oocytes and early embryos isolated from Oregon R females, (from Petrova et al., 2018). (B) Targeted LC/MS measurements of nucleotide breakdown productions in quiescent stage 14 oocytes and early embryos. (*n*=3 samples) (Petrova et al., 2018). (C) A model overview of pathways involved with nucleotide synthesis. (D) Targeted LC/MS measurements of nucleotide levels in quiescent stage 14 oocytes and early embryos. (*n*=3) (Petrova et al., 2018). (E) Oxygen consumption rate measurements of mitochondrial activity in growing egg chambers (stage 8-9), quiescent oocytes (stage 14), and early embryos (0-2 h). (eight egg chambers per well) (*n*=4). Measurements were made using a Seahorse Xe24. *P<0.05 **P<0.005. *P*-values were calculated by Student’s *t*-test. Error bars represent standard deviation. Red text denotes compounds assayed using Ellman's reagent treatment from Petrova et al. (2018).

Previous work from Petrova et al. (2018) described a shift in the cellular redox state that supports *Drosophila* embryonic growth. During the oocyte-to-embryo transition, the metabolic state becomes more oxidative, and ROS is produced during early embryogenesis. This leads to the oxidation of many proteins, and this shift in the redox state is required for development (Petrova et al., 2018). We examined the LC/MS metabolomics data from Petrova et al. (2018) and assessed metabolic pathway enrichment. We found that, while redox state changes in early embryos, nucleotide metabolism was the single most affected metabolic pathway in their data set (Fig. 2A). A fact that was not discussed in this original manuscript. In these data, we found that the elevated nucleotide breakdown we observed in quiescent oocytes is reduced (Fig. 2B). When we examined the levels of nucleotides and intermediates, we found that nucleotide levels, particularly purines, are significantly elevated in early embryos (Fig. 2D). This increase in nucleotide levels is consistent with numerous studies showing that nucleotide levels limit growth in systems ranging from bacteria to higher eukaryotes.

We examined mitochondrial oxygen consumption during this transition and found that consistent with our previous data (Hocaoglu et al., 2021), mitochondrial respiration is suppressed in quiescent stage 14 oocytes. During embryogenesis (0-4 h), however, the suppression of respiration is reversed, and mitochondrial activity rises significantly to support early development (Fig. 2E). These data suggest nucleotide breakdown and biosynthesis are coordinated with mitochondrial activity during this developmental transition (Fig. 2C). These data also indicate that nucleotide biosynthesis is likely suppressed in quiescent oocytes partly because mitochondrial activity is required to support biosynthetic processes.

**Fig. 3.**
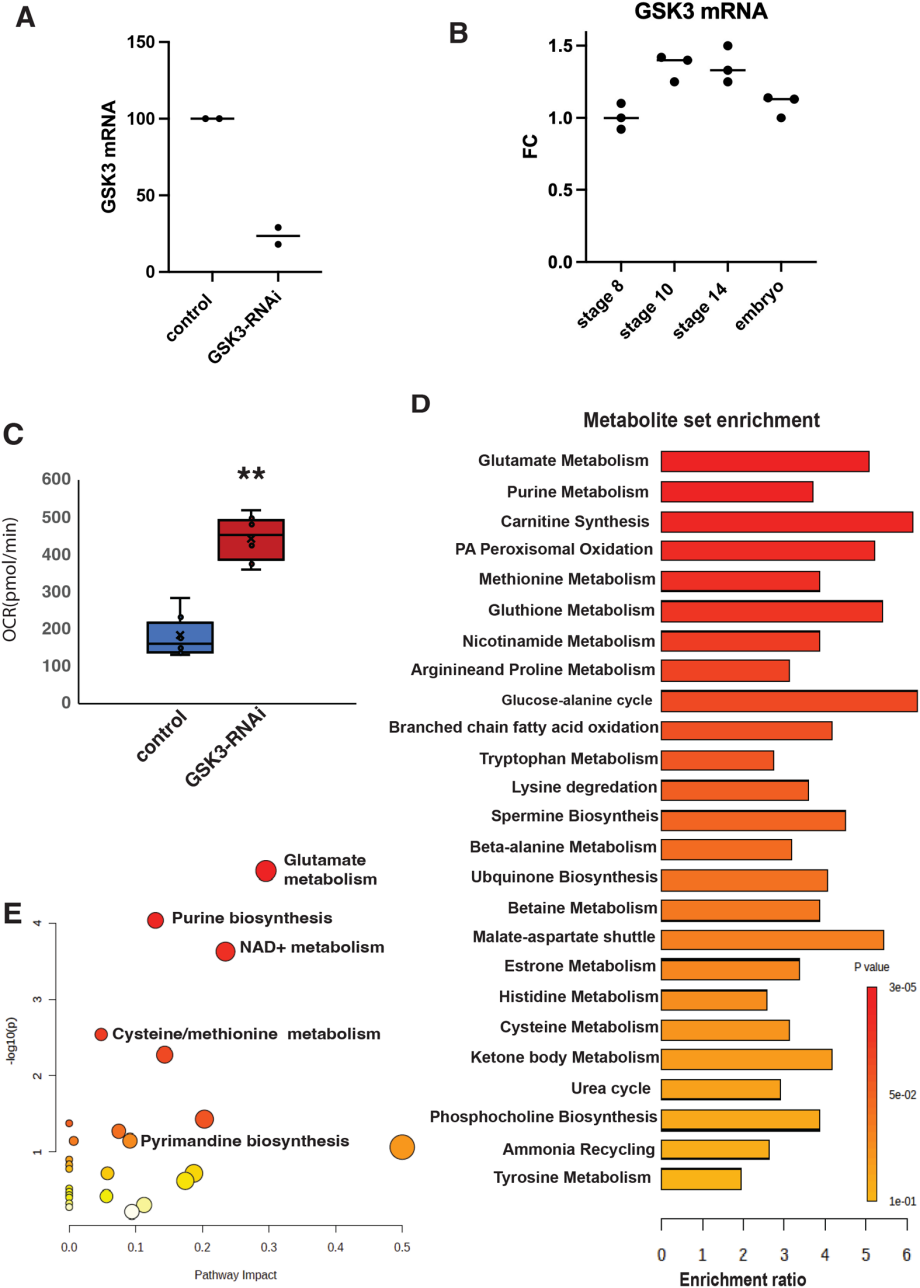
**GSK3 inhibits redox metabolism and nucleotide metabolism during oogenesis and embryogenesis.** (A) Q-PCR RNAi knockdown measurements of GSK3 mRNA levels in quiescent stage 14 oocytes from control (MTD->UAS-mCherry-RNAi) and GSK3-RNAi flies (MTD->GSK3-RNAi). (B) Q-PCR RNAi knockdown measurements of GSK3 mRNA levels in staged oocytes (8,10,14) and early embryos (4 h). (C) A violin plot showing the oxygen consumption rate (OCR) measurements of mitochondrial activity in quiescent stage 14 oocytes from control and GSK3-RNAi females. Measurements were made using a Seahorse XFp cell flux analyzer. (Eight egg chambers per well) *n*=4 samples *P<0.05 **P<0.005. *P*-values were calculated by Student’s *t*-test. (D) A bar chart showing the pathways enriched in our metabolomics analysis of stage 14 quiescent oocytes from control and GSK3-RNAi flies. (*n*=8 samples). (E) A scatter plot showing the pathway enrichment of our targeted metabolomics analysis of stage 14 quiescent oocytes from control and GSK3-RNAi flies. *n*=8 samples. Error bars represent standard deviation.

Previously, we showed that the serine-threonine kinase GSK3 is a key regulator of MRQ and redox metabolism in quiescent oocytes (Sieber et al., 2016). During the onset of quiescence, GSK3 phosphorylates targets in the mitochondrial membranes, such as VDAC and Tom40 (Yue et al., 2022). These phosphorylation events recruit the proteasome to the mitochondria and support the remodeling of the mitochondrial proteome and MRQ (Yue et al., 2022). Given the role of GSK3 in germline mitochondrial regulation and its known role as a key signaling node in the regulation of cellular metabolism, we examined whether GSK3 could be a key factor in coordinating the regulation of mitochondrial activity and nucleotide metabolism during the oocyte embryo transition. GSK3 mutant alleles cause lethality, and generating germline mutant clones would not provide enough material for LC/MS analysis. As a result, we silenced GSK3 expression in the germline using the same RNAi line (GL00277) we characterized in our previous studies of GSK3 and its role in MRQ (Sieber et al., 2016). Using MTD-GAL4, we drove GSK3 RNAi-expression, specifically in germ cells. Using Q-PCR to assess GSK3 mRNA levels, we found using this strategy effectively lowered GSK3 expression by roughly ∼80% (Fig. 3A).

We examined the oxygen consumption rate (OCR) in control and GSK3-RNAi stage 14 oocytes and found that respiration increases at least 3-fold upon GSK3 silencing (Fig. 3C). These data are consistent with our previous work showing that GSK3 suppresses electron transport chain activity in quiescent oocytes. Moreover, GSk3 expression shows a modest increase during the onset of quiescence (stages 10 and 14 of oogenesis) (Fig. 3B). To assess how GSK3 silencing impacts the metabolic profile of quiescent oocytes, we analyzed the control and GSK3-RNAi stage 14 oocytes by targeted LC/MS metabolomics. This analysis identified 48 metabolites that significantly changed in GSK3-RNAi oocytes based on a VIP score >1. Amongst those metabolites, compounds involved with redox metabolism and nucleotide biosynthesis are significantly enriched in our data set (Fig. 3D,E). In particular, glutamate metabolism, NAD+ metabolism, and purine biosynthesis are pathways highly impacted by GSK3 silencing. (Fig. 3E). These data indicate that GSK3 is crucial in coordinating mitochondrial oxidative metabolism with nucleotide homeostasis.

**Fig. 4.**
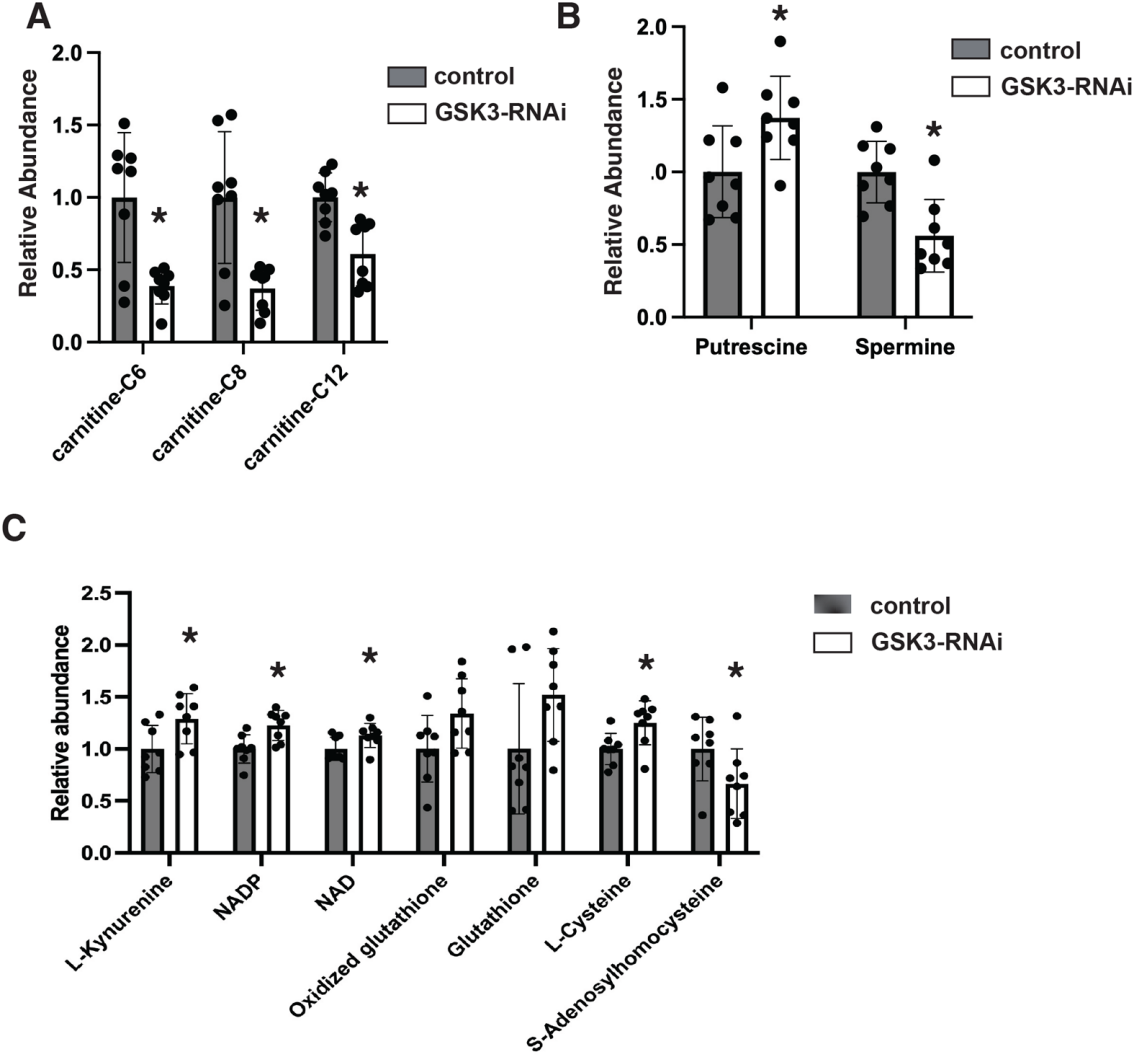
**GSK3 impacts redox metabolism and polyamine biosynthesis.** (A) LC/MS mass-spec measurements of acylcarnitine levels from control (MTD->UAS-mCherry-RNAi) and GSK3-RNAi flies (MTD->UAS-GSK3-RNAi) stage 14 oocytes. *n*=8 samples. (B) LC/MS mass-spec measurements of polyamine levels from control and GSK3-RNAi oocytes. *n*=8 samples. *VIP>1. (C) LC/MS mass-spec measurements of redox metabolite levels from control and GSK3-RNAi oocytes. *n*=8 samples. Error bars represent standard deviation. *VIP>1.

When we examined the levels of individual classes of metabolites, in line with our previous work (Yue et al., 2022), we observed a depletion of acyl-carnitines consistent with elevated fatty acid oxidation in GSK3-RNAi oocytes (Fig. 4A). This increased fatty acid catabolism likely fuels the elevated mitochondrial respiration we observed on GSK3-RNAI oocytes. Consistent with a shift in redox metabolism, we observe elevated kynurenine, NAD+, and NADP+ levels (Fig. 4C). We also observed increased levels of cysteine and glutathione synthesis that likely help maintain redox balance in the face of elevated mitochondrial activity. Interestingly, the methionine cycle intermediate S-adenosylmethionine (SAM) levels are significantly reduced, consistent with the methionine cycle feeding cysteine and glutathione biosynthesis in GSK3 -RNAi oocytes. Polyamine biosynthesis is altered, leading to increased levels of putrescene and reduced spermine levels (Fig. 4B). Polyamine biosynthesis is tightly associated with oxidative metabolism (Zahedi et al., 2022), and the block in polyamine biosynthesis we observe may stem from reduced SAM levels in GSK3-RNAi oocytes (Fig. 4C). This effect stems from SpdS and SpmS requiring SAM to convert putrescene to spermine.

**Fig. 5.**
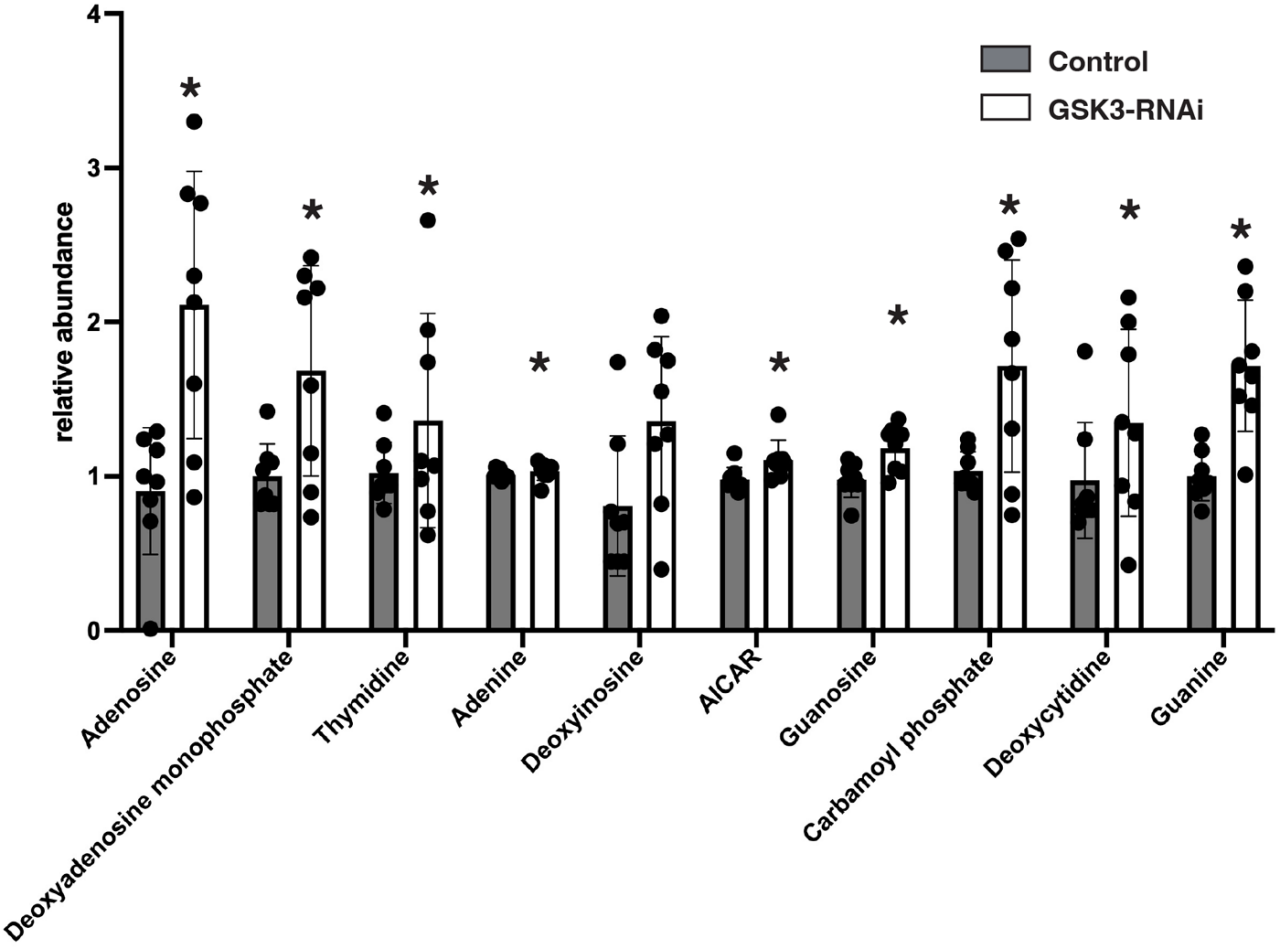
**GSK3 inhibits nucleotide metabolism and mitochondrial activity during oogenesis.** LC/MS mass-spec measurements of nucleotide levels from control (MTD->UAS- mCherry-RNAi) and GSK3-RNAi flies (MTD->GSK3-RNAi) stage 14 oocytes. *n*=8 samples. * Displays a VIP score >1 in our targeted LC/MS experiments. Error bars represent standard deviation.

In conjunction with altered levels of redox metabolites, we observed significant changes in the levels of nucleotide metabolism. Across the board, the level of most detectable nucleotides in our samples showed a significant increase (VIP score >1) (Fig. 5). These compounds include adenosine, deoxyadenosine monophosphate, thymidine, adenine, deoxyinosine, guanosine, deoxycytidine, and guanine. This increase included elevated levels of nucleotide biosynthetic intermediates AICAR and carbamoyl phosphate levels. We observed no differences in the levels of nucleotide breakdown intermediates such as hypoxanthene (Fc=0.93) and xanthene (Fc=0.98), suggesting GSK3 has a specific role in nucleotide biosynthesis (Table S2). Interestingly, seven of the nine nucleotide compounds observed changing are purines, suggesting purine biosynthesis is more sensitive to GSK3 silencing than pyrimidine production (Fig. 5). While significant in our analysis, compounds such as adenine, guanosine, and AICAR displayed milder increases, suggesting the levels of these compounds are more tightly regulated than the other nucleotides, or that mitochondrial function is affecting more distant steps in the biosynthetic process. These data show that during late oogenesis, GSK3 promotes MRQ and prevents the precocious onset of nucleotide biosynthesis seen in early embryos. These data are also consistent with recent studies showing mitochondrial defects rewire pathways with nucleotide biosynthesis in human cell lines (Wu et al., 2024).

## DISCUSSION

Each developmental stage has unique energetic and biosynthetic demands. To meet these demands, mitochondrial function must be coordinated with the production of fundamental building blocks such as nucleotides, lipids, and amino acids. Here, we show that nucleotide biosynthesis is coordinated with the reactivation of mitochondrial oxidative metabolism during the oocyte-to-embryo transition. During oocyte quiescence, mitochondrial activity is low during MRQ, and nucleotides are broken down. However, after fertilization, mitochondrial activity is restored, and cellular nucleotide levels increase dramatically. We have found that the serine-threonine kinase GSK3 is a critical factor in coordinating mitochondrial oxidative metabolism and nucleotide production. Silencing GSK3 in the germline prevents MRQ in quiescent oocytes and alters cellular redox metabolism. At the same time, GSK3 silencing leads to the precocious onset of nucleotide biosynthesis in quiescent oocytes. Our data indicates that GSK3 is a key signaling node coordinating energy production with vital biosynthetic pathways.

Biosynthetic pathways require sufficient mitochondrial activity to sustain growth. In addition to ATP, the mitochondria produce several key substrates and cofactors necessary for many biosynthetic pathways. For example, purine biosynthesis requires ATP, glutamate, and aspartate metabolism, all compounds regulated by changes in mitochondrial oxidative metabolism (Desler et al., 2007; Ahn and Metallo, 2015). Some steps of nucleotide biosynthesis even occur in the mitochondria, including aspects of single-carbon metabolism (Ducker and Rabinowitz, 2017). During quiescence and MRQ, low mitochondrial activity alters these compounds' levels and dramatically impairs the oocyte's biosynthetic potential (Sieber et al., 2016). However, once fertilized, mitochondrial function dramatically increases, providing the energy and biosynthetic capacity to support early embryonic development (Sieber et al., 2016).

Purines are proposed to be synthesized in structures called purinosomes. These microbodies are thought to contain multi-enzyme protein complexes that facilitate multiple steps in purine synthesis (Pedley et al., 2022; Zhao et al., 2013). Recent studies have suggested that purinosomes directly associate with mitochondria in growing cells. Moreover, this co-localization between purinosomes and mitochondria is regulated by mitochondrial activity (Sha and Benkovic, 2024; French et al., 2016). Recent studies have also shown that defective mitochondrial metabolism rewires purine metabolism, making cells more reliant on the salvage pathway (Wu et al., 2024). This suggests that mitochondrial activity may also be coordinated with purine synthesis spatially within the cell.

GSK3 is a well-known regulator of metabolism and development. It is highly responsive to nutrition and metabolic status due to its regulation by insulin signaling, making it an ideal factor for coordinating development and metabolism (Wang et al., 2022; Kim and Snider, 2011; Beurel et al., 2015). During oogenesis, insulin signaling is suppressed during late oogenesis (Sieber et al., 2016), leading to a GSK3-mediated suppression of mitochondrial activity. However, in the face of metabolic stress, insulin signaling precociously turns off, and GSK3 triggers a premature onset of MRQ (Sieber et al., 2016). Similarly, GSK3 is a nutrient-responsive regulator that couples nutritional status and developmental timing with coordinated shifts in mitochondrial function and nucleotide metabolism. The fact that GSK3 suppresses mitochondrial function in quiescent oocytes raises the question of whether GSK3 turns off the germline metabolism or if GSK3 prevents the precocious onset of an embryonic metabolic program. Nucleotide levels are relatively low in growing oocytes, and purine levels do not change significantly during quiescence. However, nucleotide levels increase considerably during early embryogenesis (Fig. 2). When we inhibit GSK3, we see significant increases in nucleotide levels that coincide with elevated levels of mitochondrial activity. Taken together, our data supports the model where GSK3 induces mitochondrial suppression during quiescence while at the same time preventing the precocious activation of the nucleotide biosynthesis program typically seen after fertilization in early embryos.

## MATERIALS AND METHODS

### Fly stocks

1. Oregon R.
2. y^1^ sc* v^1^ sev^21^; UAS-GSK3-RNAi (GL00227)attP2/TM3, Sb^1^ (BDSC#35321).
3. y^1^ sc* v^1^ sev^21^; UAS-mCherry-RNAi}attP2 (BDSC#35785).
4. “MTD-GAL4” P{otu-GAL4::VP16.R}1, w*; P{GAL4-nanos.NGT}40; P{GAL4::VP16-nanos.UTR}CG6325^MVD1^ (BDSC# 31777).

### Fly culture conditions

Flies were grown on standard molasses media containing 336 g agar, 630 g yeast, 2520 g cornmeal, 3360 ml molasses, and 33.4 l of H_2_O. Antifungals (Tegosept and propionic acid) were added after food has cooled to 60°C. All fly stocks are maintained at 18°C. All crosses and experiments were grown at 25°C with 50% humidity unless otherwise specified.

### Oocyte collection

Flies were fed yeast paste and added to standard media for 48 h. Ovaries were then dissected and disassociated into individual egg chambers in Grace's insect media (Gibco, #11605094). Samples of 300 stage-14 egg chambers were collected and flash-frozen using liquid nitrogen for subsequent metabolomics studies.

### Targeted LC/MS metabolomics

Samples of 300 stage 14 oocytes (10 mg/sample) were washed three times with fresh 1xPBS and then flash-frozen in liquid nitrogen. Samples were weighed and then homogenized in 1 ml of methanol: H_2_O (80:20). Samples were vortexed for 2 min and then centrifuged ∼20,000×***g*** for 15 min. The resulting supernatant was dried down by a low-temperature speed vac. The resulting dried samples were then frozen at −80°C until analyzed. Samples are analyzed using targeted LC/MS metabolomics with the assistance of the UT Southwestern metabolomics facility. Q-TOF mass spectrometer analysis, pellets were reconstituted in 0.1% formic acid in water and vortexed for 1 min. We then spun the samples at 20,160×***g*** at 4°C for 15 min. The resulting supernatants were then loaded into auto-sampler vials for analysis. Data acquisition was performed by reverse-phase chromatography on a 1290 UHPLC liquid chromatography (LC) system interfaced to a 6550 iFunnel Q-TOF mass spectrometer (MS) (Agilent Technologies, CA, USA). The MS was operated in both positive and negative (ESI+ and ESI−) modes. Compounds are separated on an Acquity UPLC® HSS T3 column. The composition of mobile phase 1 was 0.1% formic acid in water and mobile phase B composition was 0.1% formic acid in 100% ACN. The LC gradient was 0 min: 1% B; 5 min: 5% B; 15 min: 99%; 23 min: 99%; 24 min: 1%; 25 min: 1%. The flow rate was 250 μl min^−1^. The 5 μl of sample is injected. The ESI source unit setting were as follows: gas temperature 225°C and flow 18 l min^−1^, fragmentor voltage 175 V, sheath gas temperature 350°C and flow 12 l min^−1^, nozzle voltage 500 V, and capillary voltage +3500 V in positive mode and −3500 V in negative. The instrument was set to acquire over the full *m*/*z* range of 40-1700 in both modes, with the MS acquisition rate of 1 spectrum s^−1^ in profile format. We used Profinder B.08.00 SP3 software (Agilent Technologies, CA, USA) to process our raw sample data. We have an in-house database containing retention time and accurate mass information on 600 standards from the Mass Spectrometry Metabolite Library (IROA Technologies, MA, USA) to identify peaks in our data. Using this approach, we routinely measure the abundance of ∼400 compounds in our targeted LC/MS studies. All abundances are calculated by measuring the area under the peak. Once processed by the core facility, we analyze the data using the following pipeline. Using Sciex SIMCA software, we analyzed these data sets and performed Partial Least Square (PLS) analysis to examine sample clustering. From the PLS analysis, we identify metabolites exhibiting a high VIP score (>1.0) as candidate compounds contributing to the differential clustering. Subsequent pathway impact analysis and enrichment analysis are done using MetaboAnalyst software 6.0. These data are compiled from eight independent samples unless otherwise specified in the figures or legends.

### Seahorse measurements

Ovaries are dissected and dissociated, and staged oocytes are collected. Samples of eight staged oocytes or embryos are loaded into a Seahorse assay plate containing 200 µl of grace insect media (Millipore/Sigma, cat. #G8142) (supplemented with 10 µg/ml Insulin Millipore/Sigma, cat. #19278). Samples are washed with fresh media and incubated in the assay plate for 90 min to acclimate the tissue to the media. All measurements of basil respiration are made in Grace’s Insect Medium with insulin supplemented. Each sample is measured four times, and the average is used for the basil OCR for that biological sample using either a Seahorse XFp or an Xe24. Mitochondrial-specific respiration was determined by injecting rotenone (2 µM) (Millipore/Sigma, cat. #8875) and antimycin A (2 µM) (Millipore/Sigma, cat. #A8674) and waiting 20 min before measuring OCR. These measurements establish non-mitochondrial oxygen consumption that is subtracted from the total to calculate mitochondrial oxygen consumption.

### Statistics

For pairwise experiments, *P*-values were calculated using a Student's *t*-test. For experiments requiring multiple comparisons, *P*-values were calculated using one-way ANOVA. All statistics were calculated using GraphPad Prism.

### Graphic software

All graphic models presented in this manuscript were made using Biorender (license #FH241GMNIX).

## Supplementary Material

10.1242/biolopen.061815_sup1 Supplementary information

Table S1.

Table S2.

## References

[BIO061815C1] Ahn, C. S. and Metallo, C. M. (2015). Mitochondria as biosynthetic factories for cancer proliferation. *Cancer Metab.* 3, 1. 10.1186/s40170-015-0128-2 25621173 PMC4305394

[BIO061815C2] Alaynick, W. A., Kondo, R. P., Xie, W., He, W., Dufour, C. R., Downes, M., Jonker, J. W., Giles, W., Naviaux, R. K., Giguere, V. et al. (2007). ERRgamma directs and maintains the transition to oxidative metabolism in the postnatal heart. *Cell Metab.* 6, 13-24. 10.1016/j.cmet.2007.06.007 17618853

[BIO061815C3] Beurel, E., Grieco, S. F. and Jope, R. S. (2015). Glycogen synthase kinase-3 (GSK3): regulation, actions, and diseases. *Pharmacol. Ther.* 148, 114-131. 10.1016/j.pharmthera.2014.11.016 25435019 PMC4340754

[BIO061815C4] Chi, C., Ronai, D., Than, M. T., Walker, C. J., Sewell, A. K. and Han, M. (2016). Nucleotide levels regulate germline proliferation through modulating GLP-1/Notch signaling in C. elegans. *Genes Dev.* 30, 307-320. 10.1101/gad.275107.115 26833730 PMC4743060

[BIO061815C5] Desler, C., Munch-Petersen, B., Stevnsner, T., Matsui, S., Kulawiec, M., Singh, K. K. and Rasmussen, L. J. (2007). Mitochondria as determinant of nucleotide pools and chromosomal stability. *Mutat. Res.* 625, 112-124. 10.1016/j.mrfmmm.2007.06.002 17658559

[BIO061815C6] Diehl, F. F., Miettinen, T. P., Elbashir, R., Nabel, C. S., Darnell, A. M., Do, B. T., Manalis, S. R., Lewis, C. A. and Vander Heiden, M. G. (2022). Nucleotide imbalance decouples cell growth from cell proliferation. *Nat. Cell Biol.* 24, 1252-1264. 10.1038/s41556-022-00965-1 35927450 PMC9359916

[BIO061815C7] Djabrayan, N. J., Smits, C. M., Krajnc, M., Stern, T., Yamada, S., Lemon, W. C., Keller, P. J., Rushlow, C. A. and Shvartsman, S. Y. (2019). Metabolic regulation of developmental cell cycles and zygotic transcription. *Curr. Biol.* 29, 1193-1198.e5. 10.1016/j.cub.2019.02.028 30880009 PMC6501590

[BIO061815C8] Du, Y., Gupta, P., Qin, S. and Sieber, M. (2023). The role of metabolism in cellular quiescence. *J. Cell Sci.* 136, jcs260787. 10.1242/jcs.260787 37589342 PMC10445740

[BIO061815C9] Ducker, G. S. and Rabinowitz, J. D. (2017). One-carbon metabolism in health and disease. *Cell Metab.* 25, 27-42. 10.1016/j.cmet.2016.08.009 27641100 PMC5353360

[BIO061815C10] French, J. B., Jones, S. A., Deng, H., Pedley, A. M., Kim, D., Chan, C. Y., Hu, H., Pugh, R. J., Zhao, H., Zhang, Y. et al. (2016). Spatial colocalization and functional link of purinosomes with mitochondria. *Science* 351, 733-737. 10.1126/science.aac6054 26912862 PMC4881839

[BIO061815C11] Hocaoglu, H., Wang, L., Yang, M., Yue, S. and Sieber, M. (2021). Heritable shifts in redox metabolites during mitochondrial quiescence reprogramme progeny metabolism. *Nat. Metab.* 3, 1259-1274. 10.1038/s42255-021-00450-3 34545253 PMC8462065

[BIO061815C12] Kim, W. Y. and Snider, W. D. (2011). Functions of GSK-3 signaling in development of the nervous system. *Front. Mol. Neurosci.* 4, 44. 10.3389/fnmol.2011.00044 22125510 PMC3221276

[BIO061815C13] Kronja, I., Yuan, B., Eichhorn, S. W., Dzeyk, K., Krijgsveld, J., Bartel, D. P. and Orr-Weaver, T. L. (2014). Widespread changes in the posttranscriptional landscape at the Drosophila oocyte-to-embryo transition. *Cell Rep.* 7, 1495-1508. 10.1016/j.celrep.2014.05.002 24882012 PMC4143395

[BIO061815C14] Leatherman, J. L. and Jongens, T. A. (2003). Transcriptional silencing and translational control: key features of early germline development. *BioEssays* 25, 326-335. 10.1002/bies.10247 12655640

[BIO061815C15] Lovett, J. A. and Goldstein, E. S. (1977). The cytoplasmic distribution and characterization of poly (A)+RNA in oocytes and embryos of Drosophilia. *Dev. Biol.* 61, 70-78. 10.1016/0012-1606(77)90342-6 411706

[BIO061815C16] Mermod, J. J., Jacobs-Lorena, M. and Crippa, M. (1977). Changes in rate of RNA synthesis and ribosomal gene number during oogenesis of Drosophila melanogaster. *Dev. Biol.* 57, 393-402. 10.1016/0012-1606(77)90224-X 406151

[BIO061815C17] Mullen, N. J. and Singh, P. K. (2023). Nucleotide metabolism: a pan-cancer metabolic dependency. *Nat. Rev. Cancer* 23, 275-294. 10.1038/s41568-023-00557-7 36973407 PMC10041518

[BIO061815C18] Pedley, A. M., Pareek, V. and Benkovic, S. J. (2022). The purinosome: a case study for a mammalian metabolon. *Annu. Rev. Biochem.* 91, 89-106. 10.1146/annurev-biochem-032620-105728 35320684 PMC9531488

[BIO061815C19] Petrova, B., Liu, K., Tian, C., Kitaoka, M., Freinkman, E., Yang, J. and Orr-Weaver, T. L. (2018). Dynamic redox balance directs the oocyte-to-embryo transition via developmentally controlled reactive cysteine changes. *Proc. Natl. Acad. Sci. USA* 115, E7978-E7986. 10.1073/pnas.1807918115 30082411 PMC6112717

[BIO061815C20] Samant, S., Lee, H., Ghassemi, M., Chen, J., Cook, J. L., Mankin, A. S. and Neyfakh, A. A. (2008). Nucleotide biosynthesis is critical for growth of bacteria in human blood. *PLoS Pathog.* 4, e37. 10.1371/journal.ppat.0040037 18282099 PMC2242838

[BIO061815C21] Sha, Z. and Benkovic, S. J. (2024). Purinosomes spatially co-localize with mitochondrial transporters. *J. Biol. Chem.* 300, 107620. 10.1016/j.jbc.2024.107620 39098527 PMC11402301

[BIO061815C22] Shore, D. and Albert, B. (2022). Ribosome biogenesis and the cellular energy economy. *Curr. Biol.* 32, R611-R617. 10.1016/j.cub.2022.04.083 35728540

[BIO061815C23] Sieber, M. H. and Spradling, A. C. (2015). Steroid Signaling establishes a female metabolic state and regulates SREBP to control oocyte lipid accumulation. *Curr. Biol.* 25, 993-1004. 10.1016/j.cub.2015.02.019 25802149 PMC6894397

[BIO061815C24] Sieber, M. H. and Spradling, A. C. (2017). The role of metabolic states in development and disease. *Curr. Opin. Genet. Dev.* 45, 58-68. 10.1016/j.gde.2017.03.002 28347941 PMC6894399

[BIO061815C25] Sieber, M. H., Thomsen, M. B. and Spradling, A. C. (2016). Electron transport chain remodeling by GSK3 during oogenesis connects nutrient state to reproduction. *Cell* 164, 420-432. 10.1016/j.cell.2015.12.020 26824655 PMC6894174

[BIO061815C26] Tennessen, J. M., Baker, K. D., Lam, G., Evans, J. and Thummel, C. S. (2011). The Drosophila estrogen-related receptor directs a metabolic switch that supports developmental growth. *Cell Metab.* 13, 139-148. 10.1016/j.cmet.2011.01.005 21284981 PMC3072597

[BIO061815C27] Tennessen, J. M., Bertagnolli, N. M., Evans, J., Sieber, M. H., Cox, J. and Thummel, C. S. (2014). Coordinated metabolic transitions during Drosophila embryogenesis and the onset of aerobic glycolysis. *G3 (Bethesda)* 4, 839-850. 10.1534/g3.114.010652 24622332 PMC4025483

[BIO061815C28] Tippetts, T. S., Sieber, M. H. and Solmonson, A. (2023). Beyond energy and growth: the role of metabolism in developmental signaling, cell behavior and diapause. *Development* 150, dev201610. 10.1242/dev.201610 37883062 PMC10652041

[BIO061815C29] Van Blerkom, J. (2011). Mitochondrial function in the human oocyte and embryo and their role in developmental competence. *Mitochondrion* 11, 797-813. 10.1016/j.mito.2010.09.012 20933103

[BIO061815C30] Wang, L., Li, J. and Di, L. J. (2022). Glycogen synthesis and beyond, a comprehensive review of GSK3 as a key regulator of metabolic pathways and a therapeutic target for treating metabolic diseases. *Med. Res. Rev.* 42, 946-982. 10.1002/med.21867 34729791 PMC9298385

[BIO061815C31] Wu, Z., Bezwada, D., Cai, F., Harris, R. C., Ko, B., Sondhi, V., Pan, C., Vu, H. S., Nguyen, P. T., Faubert, B. et al. (2024). Electron transport chain inhibition increases cellular dependence on purine transport and salvage. *Cell Metab.* 36, 1504-1520.e9. 10.1016/j.cmet.2024.05.014 38876105 PMC11240302

[BIO061815C32] Yue, S., Wang, L., Demartino, G. N., Zhao, F., Liu, Y. and Sieber, M. H. (2022). Highly conserved shifts in ubiquitin-proteasome system (UPS) activity drive mitochondrial remodeling during quiescence. *Nat. Commun.* 13, 4462. 10.1038/s41467-022-32206-2 35915093 PMC9343427

[BIO061815C33] Zahedi, K., Barone, S. and Soleimani, M. (2022). Polyamines and their metabolism: from the maintenance of physiological homeostasis to the mediation of disease. *Med. Sci.* 10, 38. 10.3390/medsci10030038 PMC9326668 35893120

[BIO061815C34] Zhao, H., French, J. B., Fang, Y. and Benkovic, S. J. (2013). The purinosome, a multi-protein complex involved in the de novo biosynthesis of purines in humans. *Chem. Commun.* 49, 4444-4452. 10.1039/c3cc41437j PMC3877848 23575936

[BIO061815C35] Zheng, X., Boyer, L., Jin, M., Mertens, J., Kim, Y., Ma, L., Ma, L., Hamm, M., Gage, F. H. and Hunter, T. (2016). Metabolic reprogramming during neuronal differentiation from aerobic glycolysis to neuronal oxidative phosphorylation. *eLife* 5, e13374. 10.7554/eLife.13374 27282387 PMC4963198

