# Supplementary material for: GSK3 coordinately regulates mitochondrial activity and nucleotide metabolism in quiescent oocytes
Source: Biol Open. 2025 Mar 24;14(3):bio061815. doi: 10.1242/bio.061815 (PMC11972070; doi:10.1242/bio.061815)
Supplement: Supplementary information [file biolopen-14-061815-s1.pdf]

Table S1.

Available for download at
https://journals.biologists.com/bio/article-lookup/doi/10.1242/bio.061815#supplementary-data

Table S2.

Available for download at
https://journals.biologists.com/bio/article-lookup/doi/10.1242/bio.061815#supplementary-data

Biology Open • Supplementary information
